# Effects of GABA/*β*-glucan supplements on melatonin and serotonin content extracted from natural resources

**DOI:** 10.1371/journal.pone.0247890

**Published:** 2021-03-05

**Authors:** Sayan Deb Dutta, Dinesh K. Patel, Keya Ganguly, Ki-Taek Lim

**Affiliations:** Department of Biosystems Engineering, Interdisciplinary Program in Smart Agriculture, Institute of Forest Sciences, Kangwon National University, Chuncheon, Republic of Korea; Kyungpook National University, REPUBLIC OF KOREA

## Abstract

**Objective:**

This study aimed to monitor the secretion of serotonin and melatonin in the blood serum of rats in the presence of rice bran (RB), and *Sarcodon aspratus* (S) extracts for sleep promotion.

**Background:**

Sleep is a natural physiological phenomenon, and sleep disorders may cause severe mental hazards leading to excessive daytime sleepiness (EDS). The *γ*-aminobutyric acid (GABA) and *β*-glucan are the essential active ingredients of RB and mushroom, respectively, exhibited stress-reduction and nerve stabilizing potential through regulation of melatonin and serotonin hormones.

**Methods:**

Cytotoxicity of the extracts (RBS) was evaluated through WST-1 assay. The melatonin and serotonin concentrations in the blood serum were measured through ELISA kits. The Ig ELISA kit measured the immunoglobulin’s (IgG, IgM, and IgA) concentrations.

**Results:**

Improved cell viability was observed in RBS treated groups than control, indicating their biocompatibility. The melatonin and serotonin levels were high in RBS (5:5 and 7:3) treated groups compared to the control. Enhanced expression of immunoglobulin (Ig) A and G level was observed in RBS treated rats. The serotonergic genes (5-HTT, 5-HT 1B, and MAO-A) expression levels were upregulated in RBS treated groups vis-à-vis the control.

**Conclusion:**

Based on these results, we anticipated that RBS supplements could promote the sleep phenomenon by elevating the serotonin/melatonin level in the blood through the serotonergic system. Therefore, RBS supplements can be utilized as functional food material for sleep promotion.

## Introduction

Sleep is the fundamental physiological function of the human body and is required to maintain the critical roles of humans. It helps the restoration of physical or mental fatigue in the living organism [1]. The mental fatigue and decline in enthusiasm occurred in sleep disorder and exhibited a detrimental effect on overall disease recovery. Various health hazards, including socio-psychological phenomena like concentration and neurophysiological disorders, have also been observed in sleep disorders [2]. Insomnia is a kind of physiological disorder responsible for improper sleeping and causing dizziness during working times [3,4]. It is well-known that sleep is closely related to the immunity of the living organism. The central nervous system (CNS) is directly related to the immune response *via* autocrine and neuroendocrine signaling pathways to regulate immune functions. The excitatory neurotransmitters and essential hormones are produced and released by specific pathways and interacted with immune cells to produce cytokines [5]. Sedative agents, like GABA_A_-benzodiazepine (BZD) receptor agonists (muscimol, gaboxadol) and histamine H_1_ receptor (H_1_R) agonists (loratadine), are commonly explored for the treatment of insomnia. However, the prolonged consumption of these hypnotic drugs may cause adverse effects on the liver, spleen, kidney, and other disorders [6]. Therefore, it is necessary to develop complementary food products and herbal drugs, which can be utilized as natural sedative agents to treat insomnia and minimize the side effects of the hypnotic drugs.

Extracts from natural sources have received a considerable amount of attention for the treatment of sleep-related disorders due to their safety and efficiency [7]. The sleep-promoting potential has been observed in the extracts of rice bran, valerian, hops, false vine, semen zizyphi, green tea, ginseng, and ashwagandha [7–9]. Rice bran (RB), a by-product of rice is obtained from the rice milling process. RB contains vitamin E, *γ*-oryzanol fractions, and can stimulate osteo-anagenesis and promote cell proliferation as well as protein synthesis [10]. *γ*-aminobutyric acid (GABA) is an important and active component of RB, which showed the neuro-stabilizing potential [11,12]. *Sarcodon aspratus* (Neungee) mushroom is well-known for its preventive and therapeutic properties and is commonly taken as food in Korea and Japan. It is a rich source of vitamin B, ergosterol, essential amino acids, carbohydrates and *β*-glucan. It has been observed that *β*-glucan can reduce stress and mental problems by controlling the immune system [13].

Herein, we investigated the synergistic effects of RB and *S*. *aspratus* (RBS) extracts for the production of sleep-promoting neurotransmitters in the blood serum of rats. Cytotoxicity of RBS supplements was evaluated in the presence of human mesenchymal stem cells (hMSCs) after 24 h of treatment. The effects of RBS supplements on serotonin/melatonin secretion were also evaluated. The 7:3 RBS supplements exhibited the greater secretion of sleep-inducing biomarkers (serotonin and melatonin) compared to the other compositions (3:7, and 5:5) and control. It is well-known that mushrooms are rich sources of *β*-glucan, which has therapeutics and immunomodulatory potential. The expression of different immunoglobulin (Ig) was also evaluated in the presence of RBS supplements.

## Materials and methods

### Materials

RB and Neungee mushroom were obtained from the Cheorwon Agricultural Cooperative Ltd. (Cheorwon, Republic of Korea), and Kunming John Lee Mushroom Co. (Yunnan, China), respectively.

### RBS extraction

**Fig 1:**
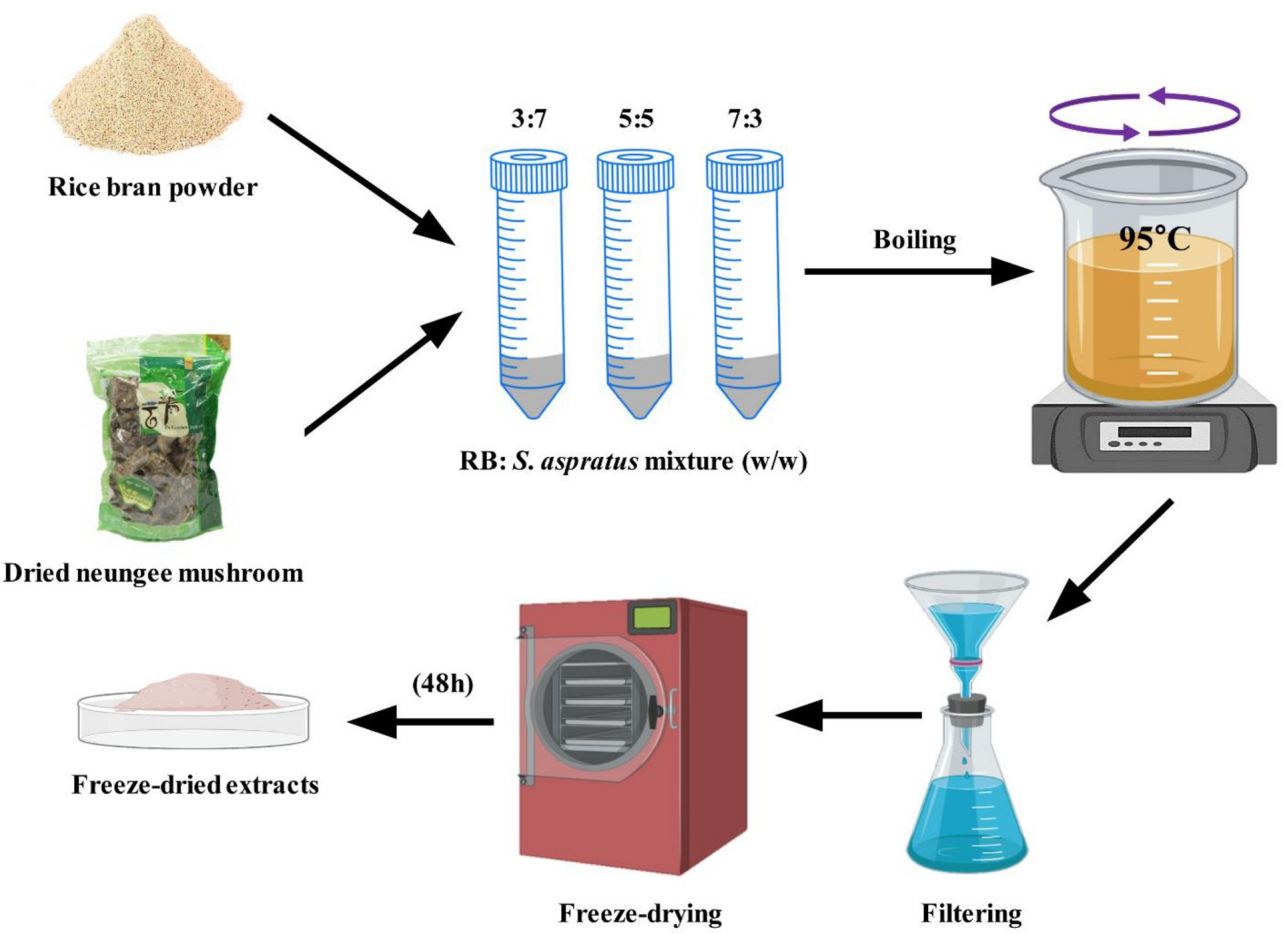
Schematic presentation for the preparation of rice bran (RB) and *Sarcodon aspratus* (Neungee) mushroom mixture (RBS supplement).

The required amounts of RB and Neungee mushroom were taken in a ratio of 3:7, 5:5, and 7:3 (w/w) and treated with double distilled water at 95°C for 1 h to obtain the RBS supplements. The treated solutions were filtered, and this process was repeated twice. The extract was concentrated by a rotary evaporator (EYELA Rotary evaporator, Japan), and freeze-dried (Freeze Dryer, EYELA^®^ Freeze Drying Unit 2200, Tokyo, Japan) for 48 h to make it in powdered form. The schematic presentation for RBS extraction from RB and Neungee mushroom is shown in **Fig 1**.

### *γ*- aminobutyric acid (GABA) content

The content of GABA in RBS supplements was quantified as earlier described somewhere else [14]. Briefly, 0.1g of each extract (3:7, 5:5, and 7:3) was added into 400 μL of methanol solution, and heated the mixture on a water bath at 75°C for 30 min, followed by the incorporation of 1 mL of 70 mM lanthanum chloride (LaCl_3_). The solution was centrifuged at 15,000 rpm for 15 min, and the supernatant was separated from the mixture. The supernatant solution (700 μL) was treated with 160 mL of 0.1 M potassium hydroxide (KOH) (Sigma-Aldrich, USA) and centrifuged at 15,000 rpm for 10 min and filtered. After this, the 200 μL of 0.5 M potassium pyrophosphate (K_4_P_2_O_7_), 150 μL of 4 mM nicotinamide adenine dinucleotide phosphate (NADP), and 50 μL of GABase (2 U/mL; Sigma-Aldrich, USA) were added into supernatant (550 μL), and absorbance was measured at 340 nm (A_1_). The reaction was quenched by the addition of 50 μL of 20 mM *α*-ketoglutaric acid sodium salt (Sigma-Aldrich, USA), and the absorbance was measured at 340 nm (A_2_). The amount of GABA was calculated as the difference between A_1_ and A_2_.

### *β*-glucan content

*β*-glucan content in RBS extracts (3:7, 5:5, and 7:3) was estimated by using a mixed linkage (1,3:1,4) *β*-glucan kit (Megazyme Inc., USA) according to the manufacturer’s instructions. The amount of *β*-glucan was calculated by measuring the difference between total glucan and *α*-glucan. All samples were triplicated for 100 mg to determine *β*-glucan contents.

### Cell culture and maintenance

The hMSCs were received from the Korean Cell Line Bank (KCLB), College of Medicine, Seoul National University, Republic of Korea. The cells were cultured according to the previously described process [15–18]. Briefly, the cells were treated with Dulbecco’s modified Eagle medium (DMEM) (Welgene Inc., Republic of Korea) containing 10% fetal bovine serum (FBS) (Welgene Inc., Republic of Korea), and 1% antibiotic-antimycotic (Anti-Anti; 100X, Gibco, USA) containing penicillin (10000 units/mL), streptomycin (10000 μg/mL), and amphotericin B (25 μg/mL) at 37°C in a humidified 5% CO_2_ incubator (Thermo-Fischer Scientific, USA). The old media were replaced with fresh media after three days. After confluency (~ 80%), the cells were detached, counted, and passaged with 1 mL of 0.25% trypsin ethylenediaminetetraacetic acid (EDTA) (Gibco, USA) solution. Passages three were used in this study.

### Cell viability

The WST-1 assay process was used to evaluate the biocompatibility of RBS supplements. For this, the cells (1 × 10^4^) were seeded in 96-well plates and cultured with RBS or without RBS supplements for 24 h. The media without RBS treatment were taken as control. After this, the WST-1 dye was added in each well and further incubated for 2 h to produce the formazan. The formed formazan was quantified by a spectrophotometer (Infinite^®^ M Nano 200 Pro; TECAN, Switzerland) with an absorbance value of 450 nm (625 nm as a reference value). All experiments were performed in triplicate fashion (*n* = 3), and data are presented at mean OD ± standard deviations (SD).

### RNA isolation and real-time PCR

The RNA isolation and real-time PCR were performed as earlier described somewhere else [18,19]. In brief, the cells (4 × 10^4^) were seeded in a 24-well plate and cultured in DMEM medium with or without RBS (7:3) extracts at 37°C for 5 and 10 days. The cultured media without RBS supplements were considered as control. The total RNA was extracted by TRIzol^®^ reagent (Thermo-Fischer Scientific, USA) according to the manufacturer’s instructions. The cDNA was synthesized from the isolated RNA by reverse transcriptase (Superscript II RTase; Invitrogen, Gaithersburg, MD) and SYBR Green Master Mix (Bio-Rad, USA). The mRNA expression was quantified by qPCR using a Bio-Rad Real-Time System (CFX96^TM^ Maestro Real-Time System, Bio-Rad, USA). The reaction conditions included 39 cycles of denaturation for 15 s at 95°C and 1 min amplification at 60°C. All reactions were performed in triplicate and normalized to the housekeeping gene *β*-actin. The cycle threshold values were calculated and compared to study the control’s gene expression and RBS treated groups. The relative mRNA expression in control and treated cells was analyzed in a histogram. The specific primer sets (*β-*actin, *5HTT*, *5HT-1B*, and *MAO-A*) are listed in **Table 1**.

**Table 1:** Specific gene primers used for real-time polymerase chain reaction (qPCR).

Gene	Forward sequence	Reverse sequence
*β-actin*	GCGCAAGTACTCTGTGTGGA	ACATCTGCTGGAAGGTGGAC
*5-HTT*	TCTGAAAAGCCCCACTGGACT	TAGGACCGTGTCTTCATCAGGC
*5-HT-1B*	TGGCGTCAAGCCAAAGCGGA	AACTGGGCTCGGGTCAAGCG
*MAO-A*	GGAGAAGCCCAATCTCGCAGGC	GGGAATGCACCACGGAATGGGT

**Abbreviations:** *β-actin*: Cytoskeleton *β*-protein; 5-HTT: 5-hydroxytryptamine transporter; 5-HT-1B: 5-hydroxytryptamine receptor 1B; MAO-A: Monoamine oxidase A.

### *In vivo* study

#### Animal care and maintenance

The imprinting control region (ICR) (male, 32-34g, 6 weeks old) rats were purchased from ORIENTBIO Inc. (Seongnam, Gyeonggi-do, Republic of Korea). All rats were kept in an insulated and sound-proof room at an ambient temperature of 21 ± 2°C, with a constant relative humidity of 35 ± 2% on an automatically controlled 12 h light and 12 h dark cycle (lights off at 20:00 h). Sufficient amounts of food and water have been supplied to the rats for their care. All possible efforts were taken to minimize animal suffering and less number of animals required for the production of reliable scientific data.

#### Animal treatment

The experimental rats were divided into five groups (N = 5); G1- negative control (without any treatment), G2—positive control (with valerian or GABA), G3 –RBS 3:7 (200 mg/kg), G4 –RBS 5:5 (200 mg/kg), and G5 –RBS 7:3 (200 mg/kg). In each group, three rats (*n* = 3) were taken for the experiment. Animals were daily eaten by oral intake during the night cycle. The Animal Experimental Ethical Committee approved all the procedures involved in animal care and treatment of Kangwon National University (Institute of Animal Care and Use Committee of Kangwon National University, Permission No. KW-170922-1).

#### Estimation of serum melatonin, serotonin, and immunoglobulins

The melatonin and serotonin concentrations were measured in the blood serum of rats after the seventh day of oral administration. For this, blood serum was taken in pre-chilled 1.5 mL tubes containing EDTA from RBS treated and control groups and placed in ice. The collected samples were cooled to room temperature and incubated for 20 min, followed by centrifugation at 2,500 rpm for 10 min.

**Fig 2:**
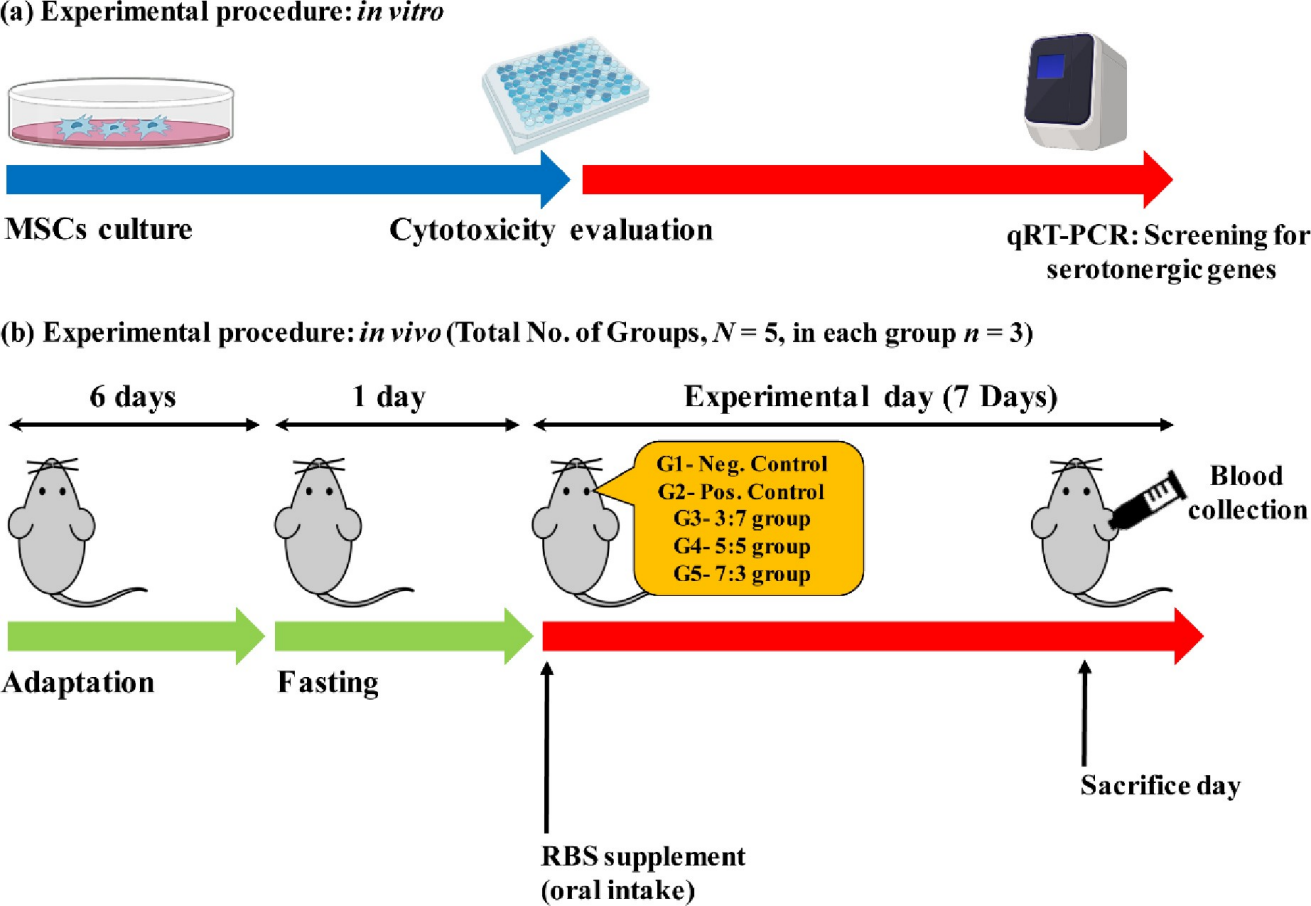
Experimental procedures and timelines for the sleep promotion test, **(a)** *in vitro* experiments, and **(b)** *in vivo* experiments.

The supernatant was transferred into a fresh tube and stored at -70°C for analysis. The melatonin and serotonin concentrations were measured by the MT ELISA kit (ELab Science, Texas, USA) and serotonin ELISA kit (Abcam, ab133053, Republic of Korea), respectively. The immunoglobulin’s (IgG, IgM, and IgA) concentrations were measured by the Ig ELISA kit (Abcam, Republic of Korea). The body weight of the experimental rats was measured at 1, day 5, and 7 days’ time intervals. All experiments were performed between 1:00 pm—4:00 pm and were fasted for 12 h before analysis. The experimental procedures and the timeline for the *in vivo* experiment are shown in **Fig 2.**

### Statistical analysis

Statistical analysis was carried out by one-way ANOVA (Origin Pro9.0) to determine the significant difference between different groups. All experiments were performed in triplicate (*n* = 3), and data are given as mean ± standard deviation (SD). Statistical significance was considered at ^*^p < 0.05.

## Results

### Quantification of GABA and *β*-glucan contents

The amounts of GABA and *β*-glucan in a different mixture of RBS supplements are given in **Table 2**. The 7:3 RBS supplements exhibited a higher content of GABA and *β*-glucan compared to the other groups. The GABA and *β*-glucan values in 7:3 RBS supplements were 0.05 ± 0.01 and 1.87 ± 0.06% (w/w), respectively. An enhancement in *β*-glucan was observed in 7:3 RBS extracts due to the presence of *β*-glucan in RB as earlier reported [19]. Therefore, an increase in GABA and *β*-glucan concentrations have occurred in RBS (7:3) supplements by the addition of RB.

**Table 2:** The γ-aminobutyric acid (GABA), and *β* glucan contents in three different mixtures of RBS supplements.

RBS (w/w)	GABA (%)	*β-*glucan (%)
3: 7	N.D.^§^	1.45 ± 0.01
5: 5	0.03 ± 0.00	1.56 ± 0.09
7: 3	0.05 ± 0.01	1.87 ± 0.06

All data shown are mean ± SD of triplicate experiments.

### Cytotoxicity evaluation

**Fig 3:**
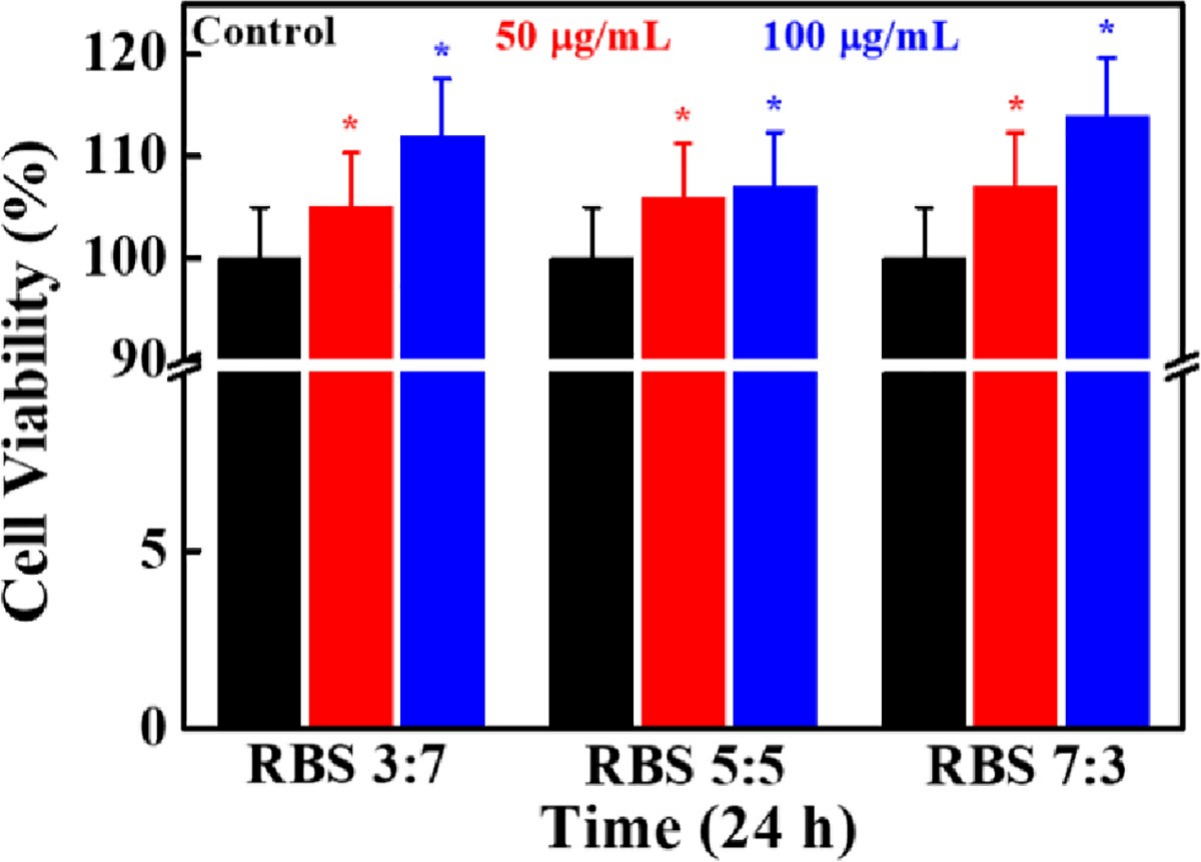
Cytotoxicity evaluation of hMSCs in the presence of RBS supplements by WST-1 assay after 24 h of treatment (*p < 0.05).

The cytotoxicity of RBS supplements was evaluated by WST-1 assay in the presence of hMSCs after 24 h of treatment, and the result is shown in **Fig 3**. The concentrations of RBS were taken at 50 and 100 μg/mL in water. Notably, better cell viability was observed in RBS treated groups compared to control, suggesting their biocompatibility. Furthermore, cytotoxicity was profoundly affected by RBS concentrations in the cultured media, and 7:3 RBS treated groups exhibited better cell viability than others. The cytotoxicity of RB was also evaluated in the presence of hMSCs at different time periods and the result is given in **S1 Fig**. No adverse effects were observed on hMSCs in the presence of RB powder, showing their biocompatibility. The cell viability was increased with increasing the concentrations of RB in the cultured media.

### Gene expression and GABA-induced serotonin signaling

**Fig 4:**
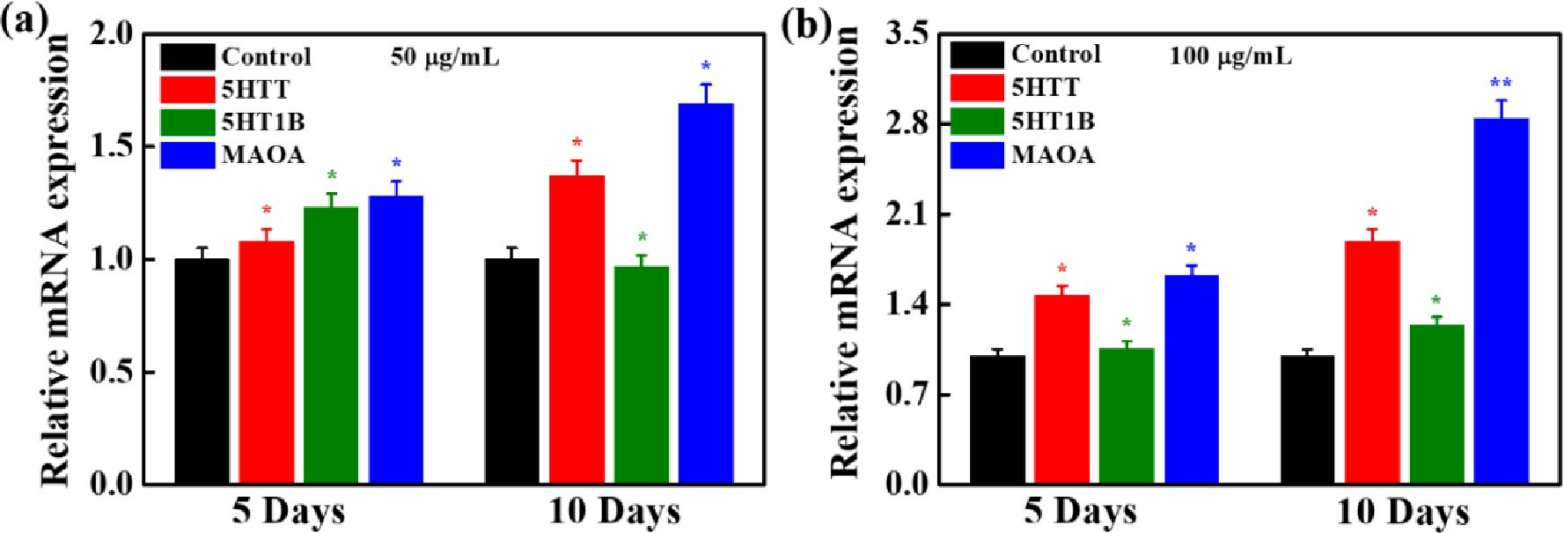
Evaluation of the effects of RBS (7:3) supplements on serotonergic associated gene markers (5HTT, 5HT-1B, and MAO-A) in hMSCs at **(a)** 50 μg/mL, and **(b)** 100 μg/mL, RBS after 5 and 10 days of treatment (*p < 0.05, & (**p < 0.01).

The expression of serotonergic genes (5HTT, 5HT-1B, and MAO-A) in hMSCs with RBS supplement (7:3) at different concentrations (50 and 100 μg/mL) was evaluated by the qPCR technique after 5 and 10 days of treatment, and the results are presented in **Fig 4**. The media without RBS supplements were considered as control. The expression of serotonergic genes was high in RBS treated media compared to control, indicating their improved serotonin expression potential. However, a decrease in the 5HT-1B gene expression occurred in RBS media after 5 days of treatment in 100 μg/mL concentration groups. The level of serotonin in the brain is a crucial factor for wakefulness and sleep.

### Effect of RBS extracts on serum serotonin and melatonin concentrations

**Fig 5:**
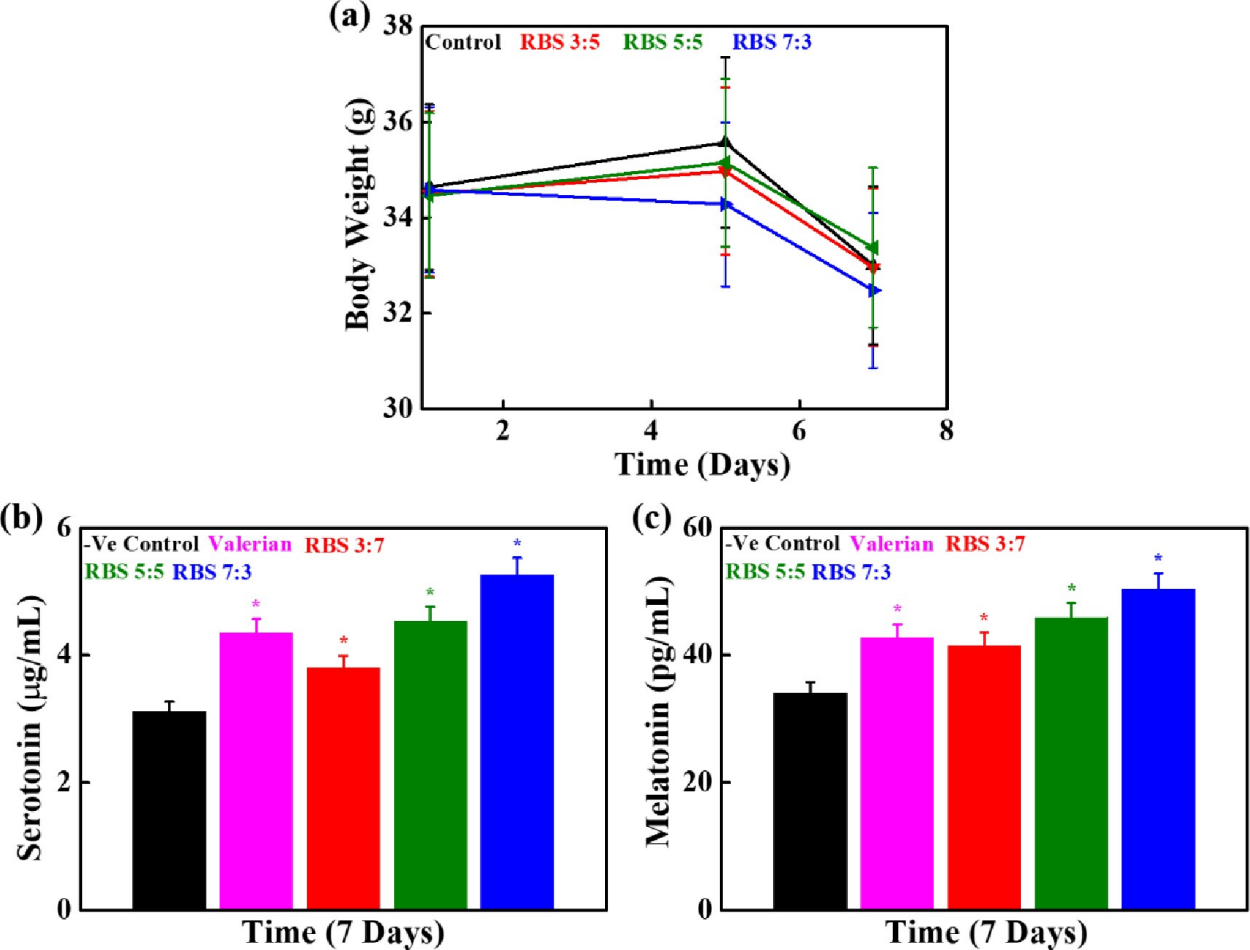
**(a)** Changes in the body weight of rats after administration of RBS supplements at indicated time intervals, **(b)** serotonin, and **(c)** melatonin concentrations in the blood serum of rats after 7 days of administration (*p < 0.05).

The changes in body weight of rats (34.54 ± 0.08g) after the oral administration of RBS supplements at different periods are shown in **Fig 5A.** The rats’ groups without treatment were considered as negative control. No significant difference in body weight was observed between the control and RBS treated groups after 7 days of the administration, suggesting that RBS supplements have no adverse effects on the rats. The changes in body weight of rats after the oral administration of pure RB powder were also monitored and the results are presented in **S2 Fig.** The rats’ groups without or with valerian treatment were considered as negative and positive control, respectively. No significant changes in the rats’ body weight were observed between the control and RB treated groups after 8 days of administration, indicating that RB has no adverse effects on the rats. No substantial change in body weight of the ICR rats was observed in the presence of mushroom as earlier reported by our groups [20]. The concentrations of serotonin and melatonin were measured in the rats’ blood serum by ELISA kit to monitor the roles of RBS supplements for sleep induction, and the results are shown in **Fig 5B & 5C.** *Valeriana officinalis-*derived valerian was used as a positive control. Valerian is commonly utilized in insomnia treatment. The serotonin concentration was 3.12 ± 0.60, and 4.35 ± 0.55 μg/mL in the negative and positive control, respectively. An elevation of serotonin concentration has occurred in the blood serum of RBS treated rats after 7 days of administration than the control. It was 4.53 ± 0.32, and 5.26 ± 0.28 μg/mL, for 5:5 and 7:3 RBS supplements, respectively. A significant increase in the melatonin concentration was also observed in RBS treated rats groups compared to the control, and its value was 45.89 ± 1.93, and 50.15 ± 0.99 pg/mL, for 5:5, and 7:3 RBS supplements, respectively. The serotonin and melatonin concentrations in blood serum of RB treated rats are given in **S1 Table.** This indicates that RBS treatment facilitates hormone production related to sleep promotion.

### Effect of RBS supplements on immunoglobulin (IgG, IgA, and IgM) concentrations

**Fig 6:**
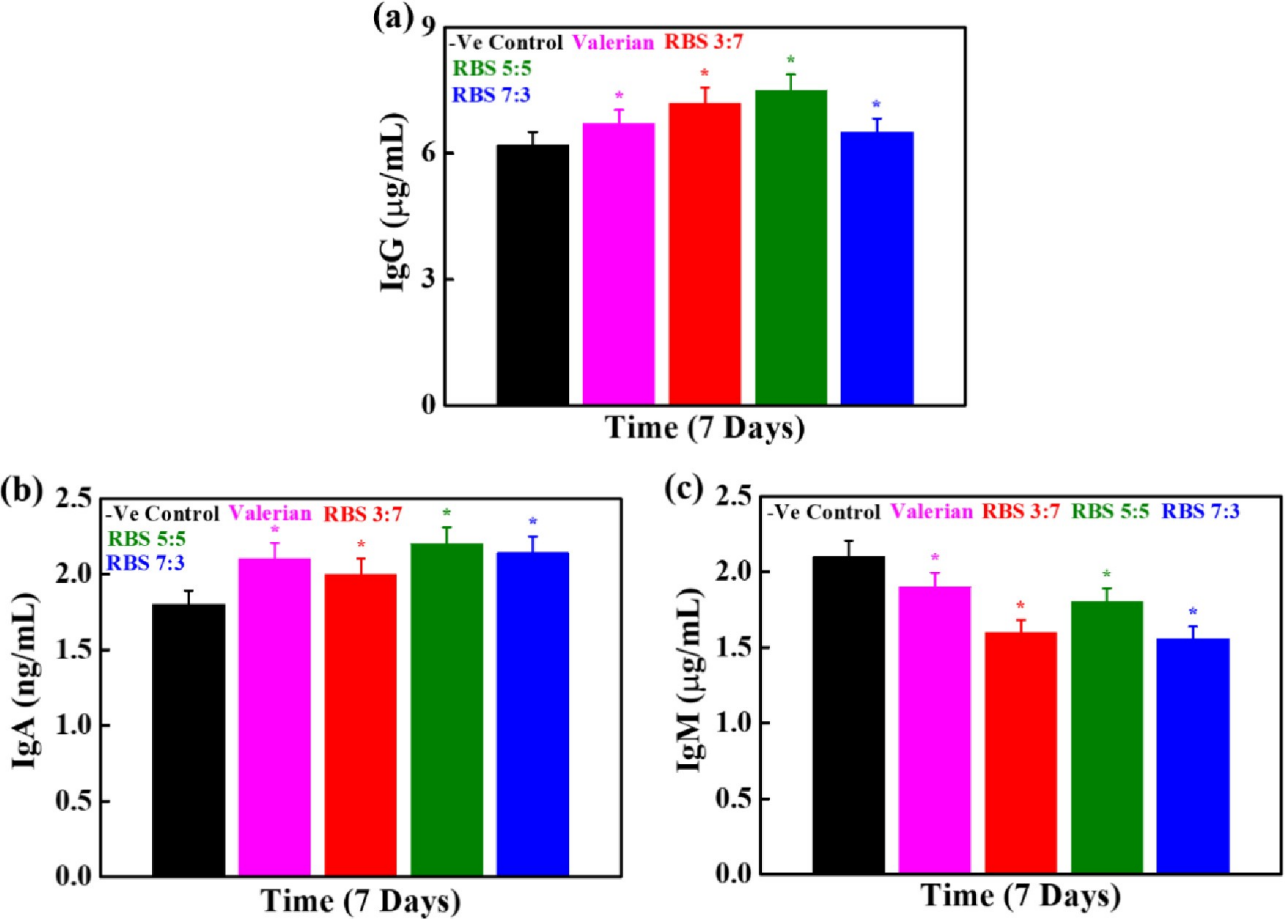
Ig concentrations in the blood serum of rats **(a)** IgG, **(b)** IgA, and **(c)** IgM after 7 days of RBS treatment (*p < 0.05).

The effects of RBS supplements on Ig production in blood serum of rats were determined by Ig ELISA kit after 7 days of treatment, and the results are given in **(Fig 6).** An enhancement in the IgG and IgA levels has occurred in RBS treated groups than control, and this was more significant in 5:5 RBS supplement than other compositions, and given in **Fig 6A & 6B.** The produced IgG and IgA levels were 7.5 ± 2 mg/mL, and 2.2 ± 2 μg/mL in 5:5 RBS supplements, respectively. However, there is no significant enhancement in IgM production occurred in RBS treated rats groups vis-à-vis control, as observed in **(Fig 6C)**. It has been reported that mushroom can alter the IgA, IgG, and IgM production, and their levels were upregulated in mushroom treated conditions compared to the control [20]. Therefore, we evaluated the combined effects of RB and mushroom on the secretion of different kinds of Ig in this study.

**Fig 7:**
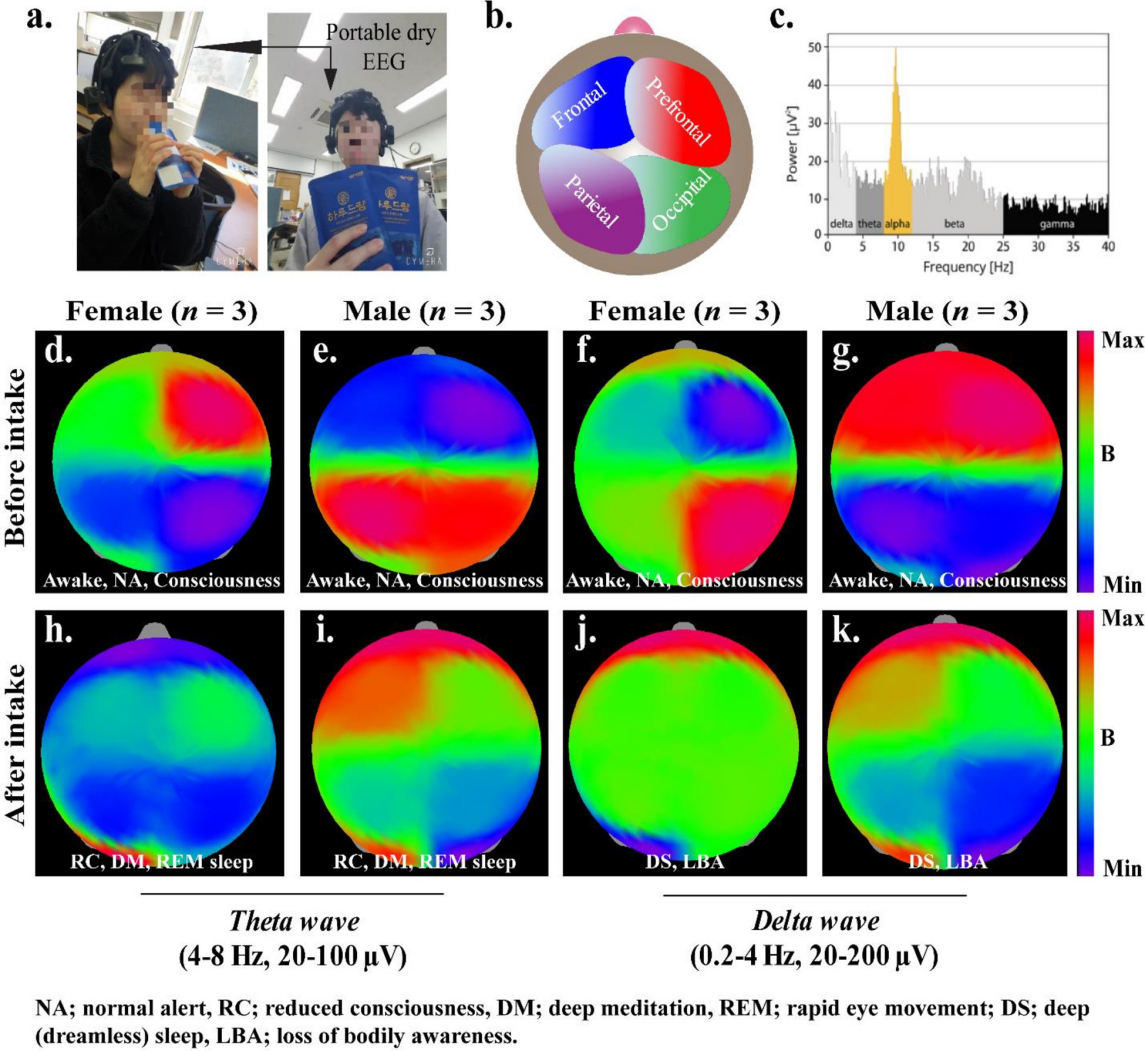
Monitoring of brain waves through the 30-Channel dry EEG headset, **(a)** wearing of the portable EEG device, **(b)** Different portions of the human brain, **(c)** frequency distribution of different brain waves, and **(d)** Generation of Theta and Delta waves after taking the commercially available Harudream^®^, which contains GABA and *β* glucan as active ingredients.

Additionally, we recorded the electroencephalography (EEG) of the commercially available Harudream^®^, which contains the mixture of both GABA and *β*-glucan to monitor the brain waves after oral intake. The position of portable EEG machine on the head of trail men/women, different positions of the human brain, various brain waves, and recorded electroencephalography are given in **(Fig 7).**

## Discussion

Vegetables, fruits, and fermented foods are the major sources of GABA. GABA occurs in the brain of the vertebrates and shows the inhibitory neurotransmitter property in the CNS. GABA plays a vital role in the physiological adjustment of pituitary gland activity and controls growth hormone (GH). Additionally, GABA reduces physiological stress and improves sleep *via* parasympathetic nerve activities [21]. *β*-glucan is a polysaccharide and linked with *β-*glycosidic bonds in the monomer unit of D-glucose. Mushrooms are enriched sources of *β*-glucan. It has immunomodulatory potential, and this activity is profoundly affected by the structure and size of *β*-glucan [20]. Enhancement in GABA and *β*-glucan concentrations was observed in RBS supplements by increasing RB amounts in the mixture. Improved cell viability has noted in RBS treated groups compared to control, suggesting their biocompatibility even at a higher concentration (100 μg/mL) of RBS supplements. Sleep plays an essential role in the maintenance of the biological clock in mammals [22]. The serotonin or 5-hydroxytryptamine (5-HT) is an extracellular multi-functional signaling molecule occurred throughout the CNS and peripheral nervous system (PNS) [23]. The melatonin is a kind of hormone secreted by the pineal gland located in the epithalamus. The pineal gland produces the serotonin and melatonin molecules and is secreted *via* a neuroendocrine pathway, modulating the sleep patterns in diurnal vertebrates, including humans. The RBS supplements were enriched in GABA and β-glucan. The biological clock regulates the rhythm of melatonin synthesis in the rat pineal gland, which occurred in the suprachiasmatic nucleus of the hypothalamus (SCN). It has been reported that neuronal activity is required to trigger melatonin synthesis, and this activity is profoundly affected by the content of GABA. The glutamatergic signaling pathway plays an important role in melatonin synthesis.

The sleep-promoting effect of exogenous melatonin has been noted in vertebrates. Yang and coworkers have studied the sleep-promoting effects and possible mechanism of RB supplements in mice by the oral administration. They observed that RB supplements decreased sleep latency and increased sleep duration in pentobarbital-induced sleep in mice [7]. It is well-known that Ig plays a significant role in the immune response against infectious diseases. Ig activates the immune response when a specific antibody recognizes an antigen. The IgA, IgG, and IgM are the isotypes of Ig and are involved in immune responses *via* strongly interacting with effectors molecules [24–26]. RB has immunomodulatory and therapeutic potential [27]. A significant enhancement in IgM and IgG production has occurred in the blood of growing pigs after the administration of rice and cassava distillers dried grains (DDG) [28]. An increase in Ig production has observed in the blood serum of RBS supplement treated rats compared to the control, showing their better immunomodulatory potential, especially in 5:5 RBS for IgA and IgG production. The 5-HT is a monoamine neurotransmitter that stimulates various physiological functions like sleep/wake cycle, thermoregulation, locomotion, food habit, blood coagulation, and cardiovascular homeostasis [29]. The synthesis of 5-HT is occurred in the intestine and acts on peripheral organs *via* G-protein-coupled transmembrane receptors (GPCR). The transport of 5-HT across the cytoplasmic membrane is mediated by 5-HT transporter (5HTT) or serotonin transporter (SERT) [29,30].

Sleep is a complex physiological process regulated by an elevated level of serotonin and melatonin neurohormones. The synthesis of 5-HT is accomplished from tryptophan *via* 5-hydroxytryptophan (5-HTP) in the CNS by serotonergic neurons. The monoamine oxidase (MAO) regulates the elevated level of serotonin. The increased level of 5-HT triggers the transcription of MAO mRNA leading to the synthesis of MAO protein, followed by the catabolization of 5-HT into 5-hydroxy indole acetic acid (5-HIAA) by MAO, and stored into the vesicles or released by the 5HT1B (rat) 5HT1B/1D (human) transporters [31]. A significant decrease in the production of 5-HT or 5-HIAA was observed during wakefulness or slow-wave sleep, and a low level of serotonin in the hypothalamus is responsible for rapid-eye-movement sleep (REM) [32,33]. Based on these results, we hypothesized a mechanism of action for RBS-induced serotonin signaling pathways for improved sleep activity. Brain activity in wakefulness and sleep is analyzed by changes in neuronal responses and rhythmic activation at different time intervals. Delta and theta waves are generated during sleeping time, and it is interesting to see that intensities of awake and consciousness waves were decreased and deep meditation and REM sleep waves were increased after taking the commercially available product, indicating their sleep-promoting activity [34]. Therefore, we also anticipated that RBS supplements have sleep promoting-potential.

**Fig 8:**
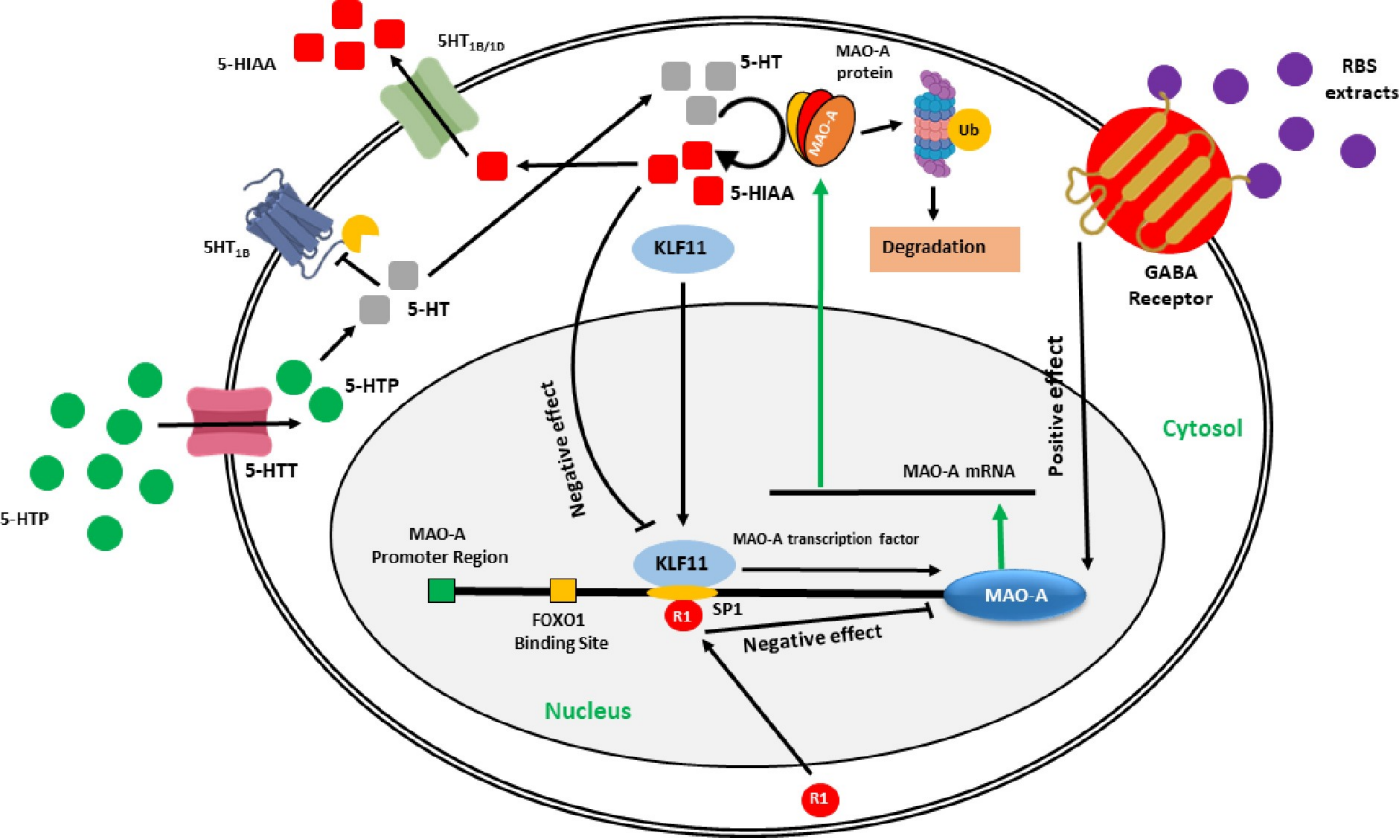
Possible mechanism of GABA-induced serotonin/melatonin signaling pathway. In CNS, 5-HT is synthesized from tryptophan *via* 5-HTP by serotonergic neurons. 5-HT is transported inside the cell by 5-HTT receptors. An elevated level of 5-HT positively triggers the gene expression of MAO-A transcription factors. Exogenous GABA is recognized by GABAergic receptors located in the cell membrane of neurons and promotes the binding of cytoplasmic transcription factors (KLF11 and R1) at the operator region of the MAO-A gene. GABA and 5-HT together trigger the early synthesis of MAO-A transcription factors *via* a positive feedback loop, resulting in the synthesis of MAO-A protein. 5-HT is then catabolized into 5-HIAA by MAO-A, and stored either in the vesicles or transported back to the peripheral blood by 5-HT1B/1D transporters. CNS: Central nervous system; 5-HT: 5-hydroxytryptamine; 5-HTTP: 5-hydroxytryptophan; 5-HTT: 5-hydroxytryptamine transporter; MAO-A: Monoamine oxidase-A; GABA: γ-aminobutyric acid; KLF11: Kruppel-like factor 11; R1: Repressor transcription factor of MAO-A; FOXO1: Forkhead box protein O1; 5-HIAA: 5-hydroxy indole acetic acid; 5-HT1B/1D: 5-hydroxytryptamine transporter 1B/1D; Ub: Ubiquitin.

The schematic presentation for RBS-induced serotonin signaling pathways for improved sleep activity is shown in **Fig 8.** The RBS supplements stimulate the transcription of the MAO-A gene *via* GABA receptor to produce elevated serotonin. However, our study is limited to observe the serotonin and melatonin levels in the blood serum of RBS treated rats, which play an important role in sleep, and EEG data from limited men/women. Furthermore, studies are required to investigate the potential roles of RBS supplements.

## Conclusion

Sleep is a physiological process of all diurnal vertebrates. The number of sleep disorder patients is increasing day-by-day globally. It was observed that the addition of RB significantly increased the GABA and *β*-glucan contents in RBS supplements. The RBS supplements have no adverse effects on hMSCs even at a higher concentration (100 μg/mL). Enhanced expression of serotonergic genes in RBS treated media by transcription of the MAO-A gene *via* the GABA receptor, suggesting its neurotransmitter production potential. No significant change in the body weight of rats has been observed in RBS treated groups compared to the control after 7 days of the oral administration. It was interesting to note that the upregulation of melatonin and serotonin neuro-hormones has occurred in the blood serum of rats in RBS treated groups vis-à-vis control. Their levels are profoundly affected by the composition of RBS supplements. These findings suggested that the proper mixture of rice bran and mushroom supplements can be utilized as naturally-derived supplements to treat sleep-related disorders.

## Supporting information

S1 Fig Cytotoxicity evaluation of human bone marrow mesenchymal stem cells (hMSCs) in the presence of rice bran (RB). All data shown are mean ± SD of triplicate experiments. Asterisks represent statistically significant differences (*p < 0.05).
(DOCX)

S2 Fig Change in body weight of rats after administration of rice bran (50, 100, and 200 mg/kg) at indicated time intervals. The values were analyzed and compared with control groups (GABA and valerian). All data shown are mean ± SD, *n* = 3 from 2 independent experiments.
(DOCX)

S1 Table The serum serotonin and melatonin concentration in the presence of rice bran (RB) extracts. (DOCX)
