# Supplementary material for: Effects of GABA/β-glucan supplements on melatonin and serotonin content extracted from natural resources
Source: PLoS One. 2021 Mar 5;16(3):e0247890. doi: 10.1371/journal.pone.0247890 (PMC7935273; doi:10.1371/journal.pone.0247890)
Supplement: S1 Fig — All data shown are mean ± SD of triplicate experiments. Asterisks represent statistically significant differences (*p < 0.05). (DOCX) [file pone.0247890.s001.docx]

*

*

*

*

*

**S1 Fig.** Cytotoxicity evaluation of RB in the presence of hMSCs at indicated time intervals (^*^*p* < 0.05).
