# Supplementary material for: Effects of GABA/β-glucan supplements on melatonin and serotonin content extracted from natural resources
Source: PLoS One. 2021 Mar 5;16(3):e0247890. doi: 10.1371/journal.pone.0247890 (PMC7935273; doi:10.1371/journal.pone.0247890)
Supplement: S2 Fig — The values were analyzed and compared with control groups (GABA and valerian). All data shown are mean ± SD, n = 3 from 2 independent experiments. (DOCX) [file pone.0247890.s002.docx]

**S2 Fig.** Change in body weight of rats after administration of rice bran (50, 100, and 200 mg/kg) at indicated time intervals. The values were analyzed and compared with control groups (GABA and valerian). All data shown are mean ± SD, *n* = 3 from 2 independent experiments.
