# Supplementary material for: Effects of GABA/β-glucan supplements on melatonin and serotonin content extracted from natural resources
Source: PLoS One. 2021 Mar 5;16(3):e0247890. doi: 10.1371/journal.pone.0247890 (PMC7935273; doi:10.1371/journal.pone.0247890)
Supplement: S1 Table — (DOCX) [file pone.0247890.s003.docx]

| Component | Concentration | | Serotonin RSD^§^ | Melatonin RSD^§^ |
| --- | --- | --- | --- | --- |
|  | Serotonin (μg/mL) | Melatonin (pg/mL) |  |  |
| RB | 45.13 ± 2.25 | 5.20 ± 0.40 | 4.98 | 7.67 |

**^§^**RSD: Relative standard deviation.

**S1 Table**. The serum serotonin and melatonin concentrations in the presence of RB supplement.
